# Supplementary material for: Audiophonologopedic Telerehabilitation: Advantages and Disadvantages from User Perspectives
Source: Children (Basel). 2024 Aug 31;11(9):1073. doi: 10.3390/children11091073 (PMC11430812; doi:10.3390/children11091073)
Supplement: Supplementary file 1 [file children-11-01073-s001.zip › children-3173432-supplementary.pdf]

__USER QUESTIONNAIRE__ (put an X for each answer)

- **You live in**:

North Italy

Centre Italy

South Italy

- **The user is a**:

Adult

Child (in this case fill in the questionnaire with a parent)

- **The pathology you are being treated for**:

Specify the pathology:

(Ex.: language delay, specific learning disorder, Down/ syndrome, cognitive deficit, dysphonia, dysphagia, aphasia…)

- **Speech therapy has begun**:

Before Covid-19 emergency in presence

Before Covid-19 emergency using remote therapy

After Covid-19 emergency using remote therapy

- **Have you already had the indispensable tools (Internet connection, computer/tablet/cell phone) to carry out telerehabilitation sessions?**

Yes, I have.

No, I had to provide as soon as possible.

- **Have you ever carried out speech telerehabilitation before Covid emergency?**

Yes, I have.

No, I have not.

If YES, for what reason?

Distance from rehabilitation centre/speech therapist

Physical inability to reach the centre

Better organization with other commitments

Use of platforms dedicated to my pathology

If NO, how have you felt about the possibility to continue therapy sessions, without interruption?

I was happy and felt relieved

I was sceptical

I was vexed about this modality

- **Type of telerehabilitation session**:

Individual

Group

Both

In case of group sessions, was it possible to continue them?

No, they have been suspended

Yes, they were carried out in telerehabilitation

- **The possibility of remote treatment:**

It was proposed by his speech therapist / Centre

It was requested by user/parent/caregiver

- **Once the first lockdown period was over (May 2020), have you maintained telerehabilitation mode?**

No, I have not. I came back in presence, because I preferred it

No, I have not. My therapist has recommended me to come back in presence

I have weekly alternated the face-to-face and distance modalities

Yes, I have

- **Weekly frequency of telerehabilitation sessions:**

Once a week

Twice a week

Three times a week

Other

- **Telerehabilitation attendance has been:**

Same as face-to-face mode

Superior to face-to-face mode

Inferior to face-to-face mode, because appointments were often skipped due to logistic problems (connection/computer availability, …)

Inferior to face-to-face mode, because I forgot some appointments

Other

- **Have you used apps, dedicated web sites or platforms for therapy sessions? (e.g. Wordwall, cognitive training, learning apps, etc.) Tick more answers if it is necessary.**

Yes, I have. (specify which ones)

Yes, I have also used them on my own, outside therapy time

No, I have not. Therapist have not proposed them

No, I have not. They are not useful for my pathology

- **Have you perceived any improvements?**

No, I have not. It was useful as maintenance therapy

Yes, I have perceived the same grade of improvements obtained in traditional therapy

Yes, I have perceived better improvements than to traditional therapy

- **According to you, which are main telerehabilitation advantages? Tick more answers if it is necessary.**

Time saving

Increased concentration

Economic factors (no public transport, parking and fuel cost, etc.)

Not having to use individual protection devices (DPI) and being able to have better feedback from therapist

Other (specify)

- **Which are the main limits of remote therapy?**

Logistic (internet connection, lack of devices, necessity of a dedicated emplacement)

Greater distractibility

Having to show on camera the activities carried out on paper

Comprehension of the exercise

Lack of comparison with other users and loss of social aspect (e.g. feeling alone with that problem)

Other (specify)

- **Have you needed help or assistance from a parent or caregiver?**

Yes, I have, but only at the beginning

Yes, I always have

No, I have not. Therapist has guided me

Never

- **In your opinion, which is the best treatment for your pathology?**

Traditional face-to-face therapy

Remote therapy
